# HIV Protease Inhibitors Sensitize Human Head and Neck Squamous Carcinoma Cells to Radiation by Activating Endoplasmic Reticulum Stress

**DOI:** 10.1371/journal.pone.0125928

**Published:** 2015-05-01

**Authors:** Runping Liu, Luyong Zhang, Jing Yang, Xiaoxuan Zhang, Ross Mikkelsen, Shiyu Song, Huiping Zhou

**Affiliations:** 1 Jiangsu Center for Drug Screening, China Pharmaceutical University, Nanjing, 210009, China; 2 Department of Microbiology & Immunology, Virginia Commonwealth University, Richmond, VA, 23298, United States of America; 3 Department of Radiation Oncology, Virginia Commonwealth University, Richmond, VA, 23298, United States of America; 4 McGuire Veterans Affairs Medical Center, Richmond, VA, 23298, United States of America; Texas A&M Health Science Center, UNITED STATES

## Abstract

**Background:**

Human head and neck squamous cell carcinoma (HNSCC) is the sixth most malignant cancer worldwide. Despite significant advances in the delivery of treatment and surgical reconstruction, there is no significant improvement of mortality rates for this disease in the past decades. Radiotherapy is the core component of the clinical combinational therapies for HNSCC. However, the tumor cells have a tendency to develop radiation resistance, which is a major barrier to effective treatment. HIV protease inhibitors (HIV PIs) have been reported with radiosensitizing activities in HNSCC cells, but the underlying cellular/molecular mechanisms remain unclear. Our previous study has shown that HIV PIs induce cell apoptosis via activation of endoplasmic reticulum (ER) stress. The aim of this study was to examine the role of ER stress in HIV PI-induced radiosensitivity in human HNSCC.

**Methodology and Principal Findings:**

HNSCC cell lines, SQ20B and FaDu, and the most commonly used HIV PIs, lopinavir and ritonavir (L/R), were used in this study. Clonogenic assay was used to assess the radiosensitivity. Cell viability, apoptosis and cell cycle were analyzed using Cellometer Vision CBA. The mRNA and protein levels of ER stress-related genes (eIF2α, CHOP, ATF-4, and XBP-1), as well as cell cycle related protein, cyclin D1, were detected by real time RT-PCR and Western blot analysis, respectively. The results demonstrated that L/R dose-dependently sensitized HNSCC cells to irradiation and inhibited cell growth. L/R-induced activation of ER stress was correlated to down-regulation of cyclin D1 expression and cell cycle arrest under G0/G1 phase.

**Conclusion and Significance:**

HIV PIs sensitize HNSCC cells to radiotherapy by activation of ER stress and induction of cell cycle arrest. Our results provided evidence that HIV PIs can be potentially used in combination with radiation in the treatment of HNSCC.

## Introduction

Human head and neck carcinoma includes a heterogeneous group of malignancies of the oral cavity, oropharynx, hypopharynx, larynx, lips, paranasal sinuses and salivary glands [1]. More than 90% of these cancers are squamous cell carcinomas (HNSCC). HNSCC represents the sixth most common malignancy worldwide [2]. The major risk factors include tobacco and alcohol consumption, poor oral hygiene, and infection by human papillomavirus (HPV) [3–5].

The majority of HNSCCs are diagnosed in locally advanced stages. Surgery, radiotherapy and chemotherapy are the current main strategies to treat HNSCC patients. Local recurrence remains the dominant pattern of treatment failure. Recent advances in the understanding of the molecular mechanisms of disease initiation and progression have led to the development of more specific therapies, such as Cetuximab, a monoclonal antibody against the epidermal growth factor receptor (EGFR). Cetuximab has been approved for combinational therapy with radiation in patients with unresectable HNSCC [6]. Overexpression of EGFR was often associated with a poor prognosis in HNSCC [7]. Although enhanced efforts have been put into effect and new therapies have been introduced during the last decade, the morbidity rate of HNSCC has not been reduced significantly [8]. A major challenge of current available therapies (radiation and chemotherapy) is the rapid development of resistance. Therefore, identification of cellular/molecular mechanisms responsible for resistance and development of new therapeutic strategies to overcome the resistance would improve the efficiency of current therapies.

Human immunodeficiency virus protease inhibitors (HIV PIs) are key components of highly active anti-retroviral therapy (HAART) for HIV infection. Previous studies from our laboratory and other laboratories demonstrated that HIV PIs are able to trigger the unfolded protein response (UPR) and endoplasmic reticulum (ER) stress [9, 10]. Three main branches of the UPR have been identified so far, including IRE1, PERK and ATF6 [11, 12]. PERK pathway activation is considered tightly correlated with cell apoptosis and survival. PERK activation-induced phosphorylation of eIF2α, a key mediator of protein translation, further disrupts translation initiation complexes and subsequently leads to global suppression of protein expression. Phosphorylation of eIF2α further induces the expression of ATF4, which leads to activation of CHOP, a proapoptotic factor [13–15]. Emerging evidence demonstrated that HIV PI-induced ER stress activation is linked to cell apoptosis in different types of cells [16–20]. Several studies reported that HIV PIs induced apoptosis through activating the STAT3/ERK1/2 pathway in human multiple myeloma cells and prostate cancer cells [21, 22]. HIV PIs are also able to induce cell cycle arrest and cell death by triggering the ER stress response [23, 24]. Recent studies further suggest that HIV PIs could be potential anti-cancer drugs due to their inhibitory effects on the PI3K-Akt signaling pathway, which is considered to be an important survival mechanism in various cancer cells [25]. Several preclinical studies indicated that down-regulation of Akt phosphorylation by HIV PI treatment or by the proteasome inhibitor, Bortezomib, resulted in increased radiosensitivity of cancer cells [20, 26, 27]. Despite the fact that HIV PIs have been identified as potential radio-sensitizers, the underlying mechanisms are still unclear. The current study was aimed at examining whether the most commonly used HIV PIs (lopinavir and ritonavir, L/R) are able to increase the radiosensitivity in HNSCC and further identify the underlying mechanisms for that increase.

## Materials and Methods

### Materials

HIV protease inhibitors, lopinavir (L) and ritonavir (R), were obtained from NIH AIDS Research & Reference Reagent Program, Division of AIDS, NIAID, NIH. Thapsigargin (TG) and dimethyl sulfoxide (DMSO) was purchased from Sigma-Aldrich (St. Louis, MO). Cell Counting Kit-8 (CCK-8) was from Dojindo Molecular Technologies (Kumamoto, Japan). Antibodies against p-ERK1/2, ERK1, ERK2, CHOP, ATP-4, XBP-1, Lamin B, p-eIF-2α, and eIF-2α were from Santa Cruz Biotechnology (Santa Cruz, CA). Mouse monoclonal antibody against β-actin and polyclonal antibodies against p-AKT, AKT, and cyclin D1 were from Thermo Scientific (Wilmington, DE). IRDye secondary antibodies were purchased from LI-COR Biosciences (Lincoln, NE). Bio-Rad protein assay reagent, Precision Plus Kaleidoscope Standards, iQTM SYBR Green Supermix were obtained from Bio-Rad (Hercules, CA). QIAzol Lysis Reagent was obtained from QIAGEN Sciences (Germantown, MD). High Capacity cDNA Reverse Transcription Kit was from Life Technologies (Grand Island, NY). Cell culture medium and supplement components were products from Invitrogen (Carlsbad, CA, USA)

### Cell culture and treatment

Human HNSCC cells, FaDu hypopharyngeal cancer cell line (HTB-43) and SQ20B cell line, were obtained from ATCC. FaDu cells were cultured in MEM medium with 10% Fetal Bovine Serum, penicillin G (100 U/ml), streptomycin (100 μg/ml) and 1% non-essential amino acids (NEAA). SQ20B cells were cultured in RPMI 1640 medium with 10% Fetal Bovine Serum, penicillin G (100 U/ml) and streptomycin (100 μg/ml). HIV PIs (L/R, 4/1 ratio) and TG were dissolved in DMSO. Drugs were directly added into the cell culture medium and incubated for different periods as described previously [28–31].

### Cell proliferation assay

SQ20B and FaDu cells were seeded into 96-well plates with complete medium and treated with HIV PIs. The viable cell numbers were determined by Cell Counting Kit-8 (CCK8), from Dojindo Molecular Technologies, Inc. (Rockville, MD). Victor^3^ Multilabel Plate Counter from Perkin Elmer (Waltham, MA) was used to measure the absorbance at 450nm as described previously [32].

### Clonogenic survival assay

SQ20B and FaDu cells were cultured in 24-well plates with or without HIV PI treatment for 24 h and followed by irradiation with different doses, 2Gy or 4Gy. After 24 h, cells were detached and plated for colony-formation assays to assess clonogenic survival. Cells were cultured for 12 days, fixed in 10% formaldehyde, and stained with 0.5% violet crystal. Colonies containing 50 or more cells were counted to determine the surviving fractions of clonogenic cells using an Olympus microscope as described previously [33].

### Annexin V and Propidium Iodide (PI) staining

SQ20B and FaDu cells were cultured in 6 well plates. After 24h treatment, cells were harvested and resuspended into a density of 10^6^cells/ml. BD Pharmingen FITC Annexin V Apoptosis Detection Kit I (BD Science, San Diego, CA) was used to perform the staining, according to the protocol recommended by the manufacturer as described previously [30]. The stained cells were then analyzed by Cellometer Vision CBA Analysis System (Nexcelom Bioscience, Lawrence, MA).

### Cell cycle assay

SQ20B and FaDu cells were treated with HIV PIs or TG or vehicle control for 24h. Cells were fixed in ice-cold methanol for 15 min and stained with a Cellometer PI Cell Cycle Kit (Nexcelom Bioscience, Lawrence,MA) according to the protocol recommended by the manufacturer. The Cellometer Vision CBA Analysis System was used to analyze the results.

### RNA isolation and quantitative real-time RT-PCR

Cells were treated with HIV PIs or vehicle control for 24 h. Total Cellular RNA was isolated using QIAzol Lysis Reagent and reverse transcribed into first-strand cDNA using a High-Capacity cDNA Transcription Kit. The mRNA levels of ATF-4, XBP-1, CHOP, and GAPDH were quantified with real-time RT-PCR as described previously [16, 17, 19, 28–30, 34, 35].

### Western Blot analysis

The nuclear proteins and total proteins were isolated as previously described [16]. The protein levels of target genes were detected using specific primary antibodies and IRDye secondary antibodies on an Odyssey Fluorescence Imaging System (LI-COR Biosciences, USA) as described previously [19, 35, 36]. The density of the immunoblots was analyzed using Odyssey V3.0 software

### Wound healing assays

SQ20B cells were plated into 12-well plates at a concentration of 5 x 10^5^ cells per well for overnight and allowed to form a confluent monolayer. After that the monolayer was scratched with a sterile pipette tip, cells were washed gently with serum free medium to remove floating and detached cells and photographed (time 0 h) using an Olympus 1x71 microscope using a 10 x objective. Cells were treated with different amounts of L/R (0–25 μM) or vehicle control (DMSO) or TG (100 nM). After 6h, the wounded area was photographed as described previously [32]. The images acquired for each treatment group were further analyzed using IPLab 4.0.

### Statistical analysis

All of the experiments were repeated at least three times and the data were expressed as the mean ± SD. One-way ANOVA was employed to analyze the differences between sets of data. To confirm the differences that occurred between groups, *post hoc* tests were used for follow-up tests. Statistics were performed using GraphPad Prism 5.0 (GraphPad, San Diego, CA) as described previously [32, 36]. A value of P<0.05 was considered statistically significant.

## Results

### Effect of HIV PIs on radiosensitivity of HNSCC cells

**Fig 1:**
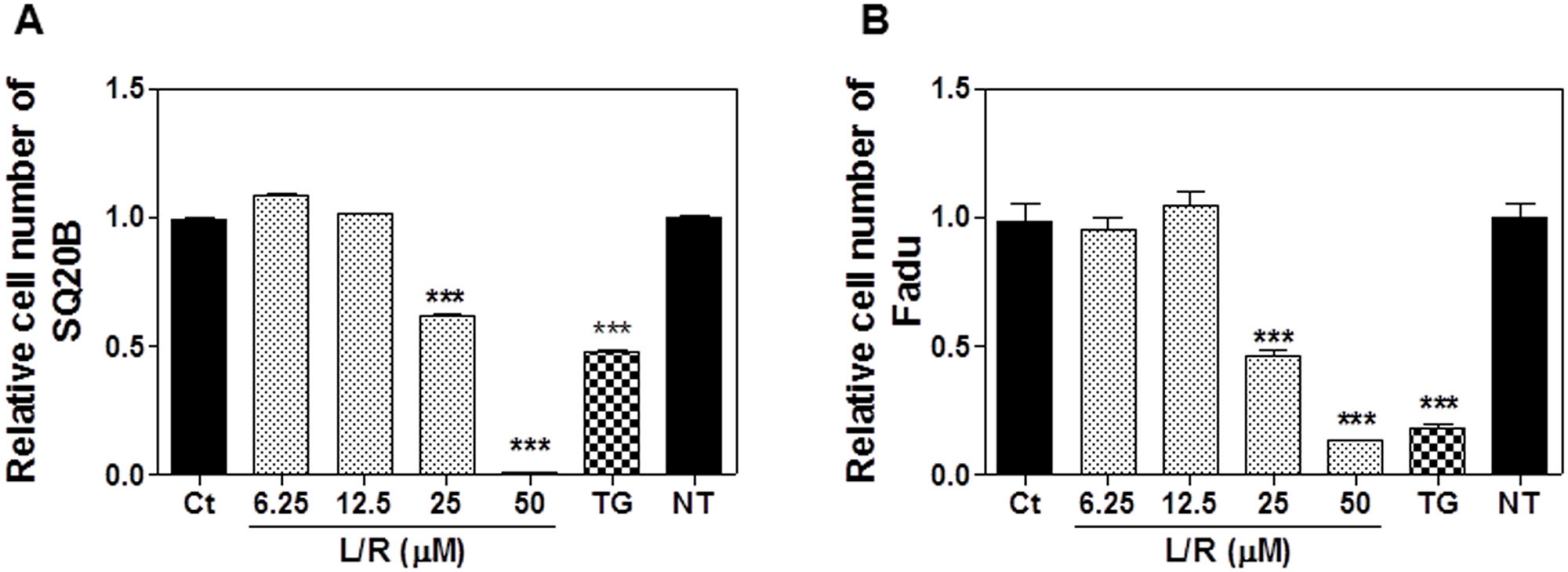
The effects of HIV PIs on HNSCC cell growth. Human HNSCC cell lines SQ20B and FaDu were cultured in complete medium and treated with different concentrations of HIV PIs (Lopinavir/Ritonavir, L/R, 0–50 μM) or Thapsigargin (TG, 50 nM) for 48h. At the end of treatment, cell viability was assessed using the CCK-8 kit according to the instructions provided by the manufacturer. Relative numbers of viable cells were calculated. (A) SQ20B; (B) FaDu. Values of each group are mean ± S.E. of six independent experiments. *** *P<0*.*001*, Statistical significance relative to control group.

**Fig 2:**
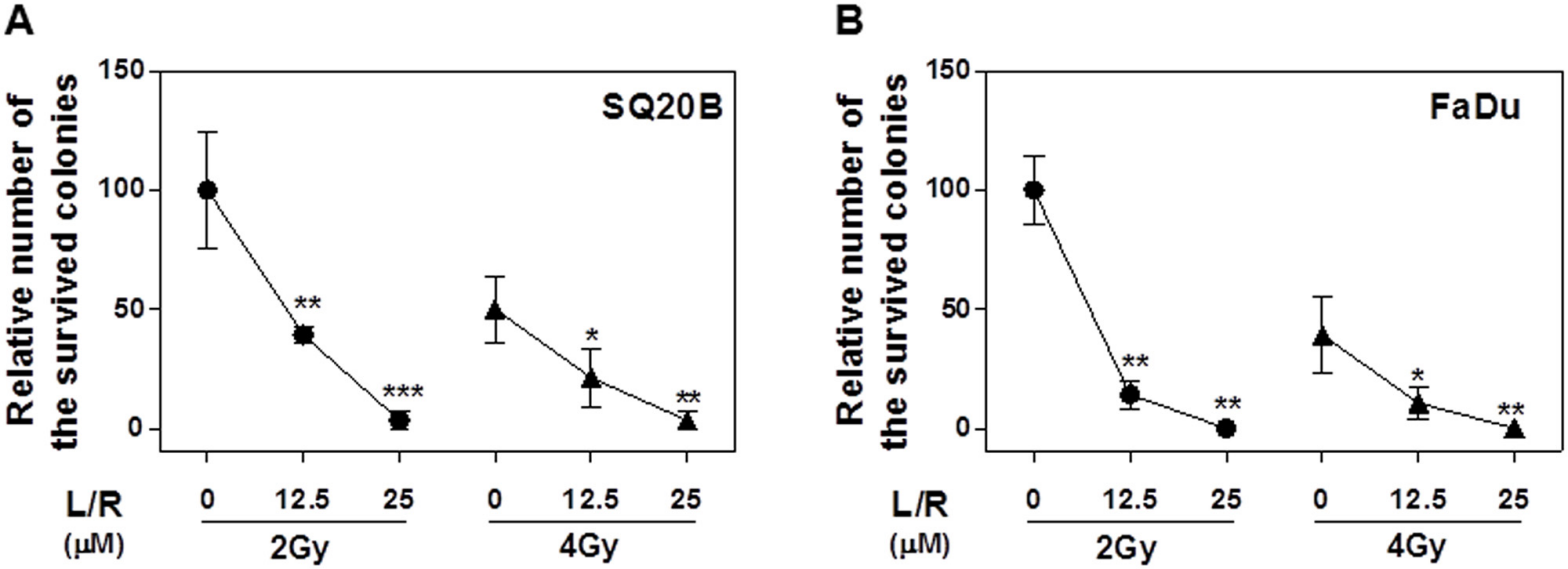
Effect of HIV PIs on radiosensitivity of HNSCC cells. Human HNSCC cells (SQ20B and FaDu) were cultured in complete medium and treated with different concentrations of HIV PIs (L/R, 12.5μM or 25μM) for 24h. At the end of treatment, cells were exposed to 2Gy or 4Gy of radiation. After irradiation, cells were detached and plated for colony-formation assays to assess clonogenic survival as described in “Methods”. Relative numbers of survival colonies are presented. (A) SQ20B; (B) FaDu; Values are mean ± S.E. of three independent experiments. ** *P<0*.*01*, *P<0.*05*, Statistical significance relative to control group.

To evaluate the effect of the most commonly used HIV PIs (L/R) on HNSCC proliferation, SQ20B and FaDu cells were treated with different concentrations of L/R (0, 6.25, 12.5, 25 or 50 μM) for 48 h. As shown in Fig 1, L/R significantly inhibited cell growth at concentrations of 25 and 50 μM in both SQ20B and FaDu cells. To further determine whether HIV PIs were able to sensitize HNSCC to radiation, SQ20B and FaDu cells were pretreated with L/R (12.5 or 25 μM) for 24 h and then irradiated at 2 Gy or 4 Gy. As shown in Fig 2, L/R significantly increased the radiosensitivity and reduced the number of survival colonies in both SQ20B and FaDu cells.

### Effect of HIV PIs on ER stress activation and cell apoptosis

**Fig 3:**
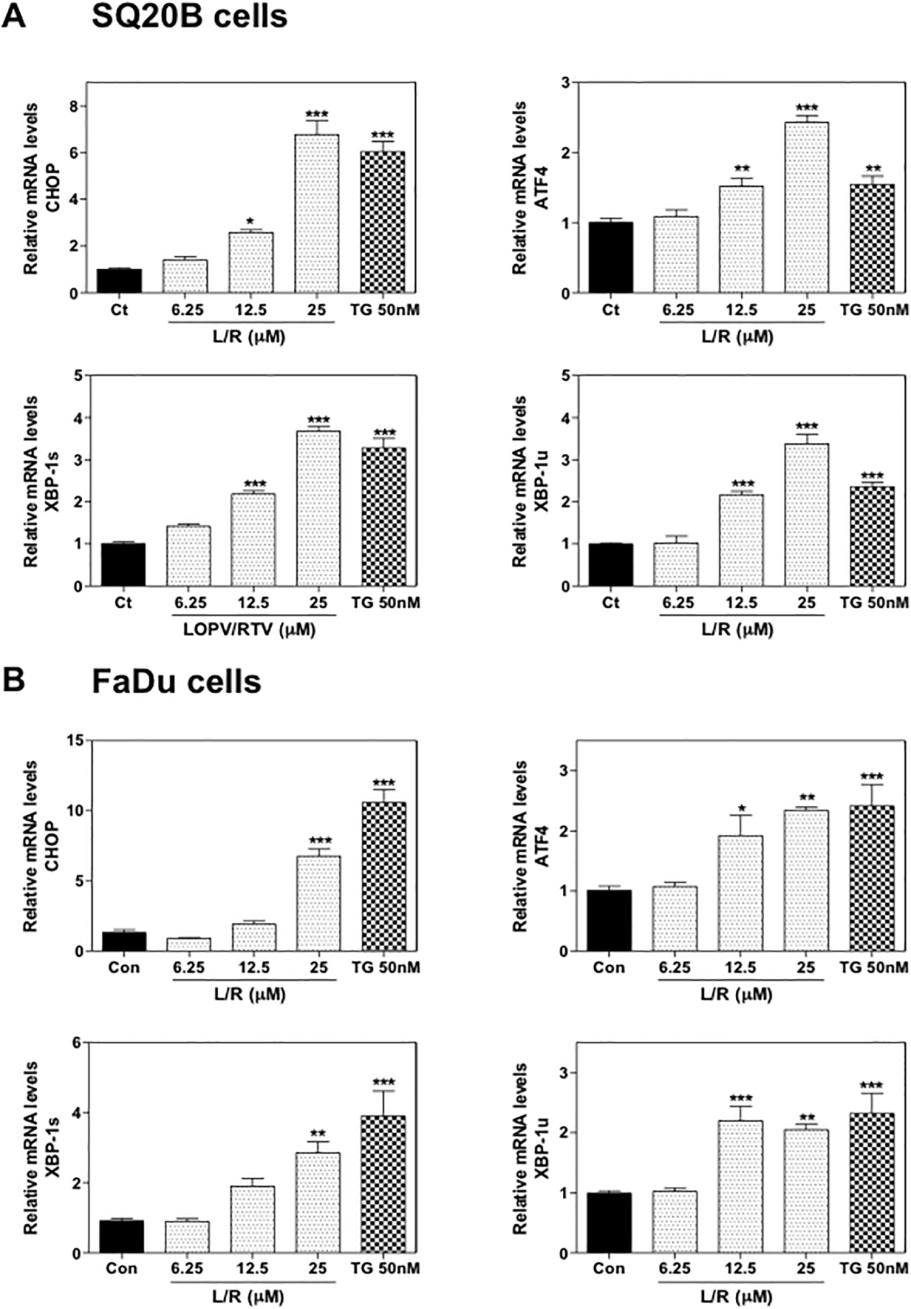
Effect of HIV PIs on mRNA levels of the UPR genes in HNSCC. HNSCC cells, SQ20B and FaDu, were cultured in complete medium and treated with different concentrations of L/R (0, 12.5, 25, and 50 μM) or TG (50 nM) for 24h. At the end of treatment, total RNAs were isolated and reverse transcribed. The relative mRNA levels of ATF-4, CHOP, spliced XBP-1 (XBP-1s) and unspliced XBP-1 (XBP-1u) were detected by real-time RT-PCR and normalized using GAPDH as an internal control as described under “Methods”. Values are mean ± S.E. of three independent experiments. Statistical significance relative to vehicle control, *P<0.*05*; **P<0.01; ***P<0.*001*. (A) SQ20B cells; (B) FaDu cells.

**Fig 4:**
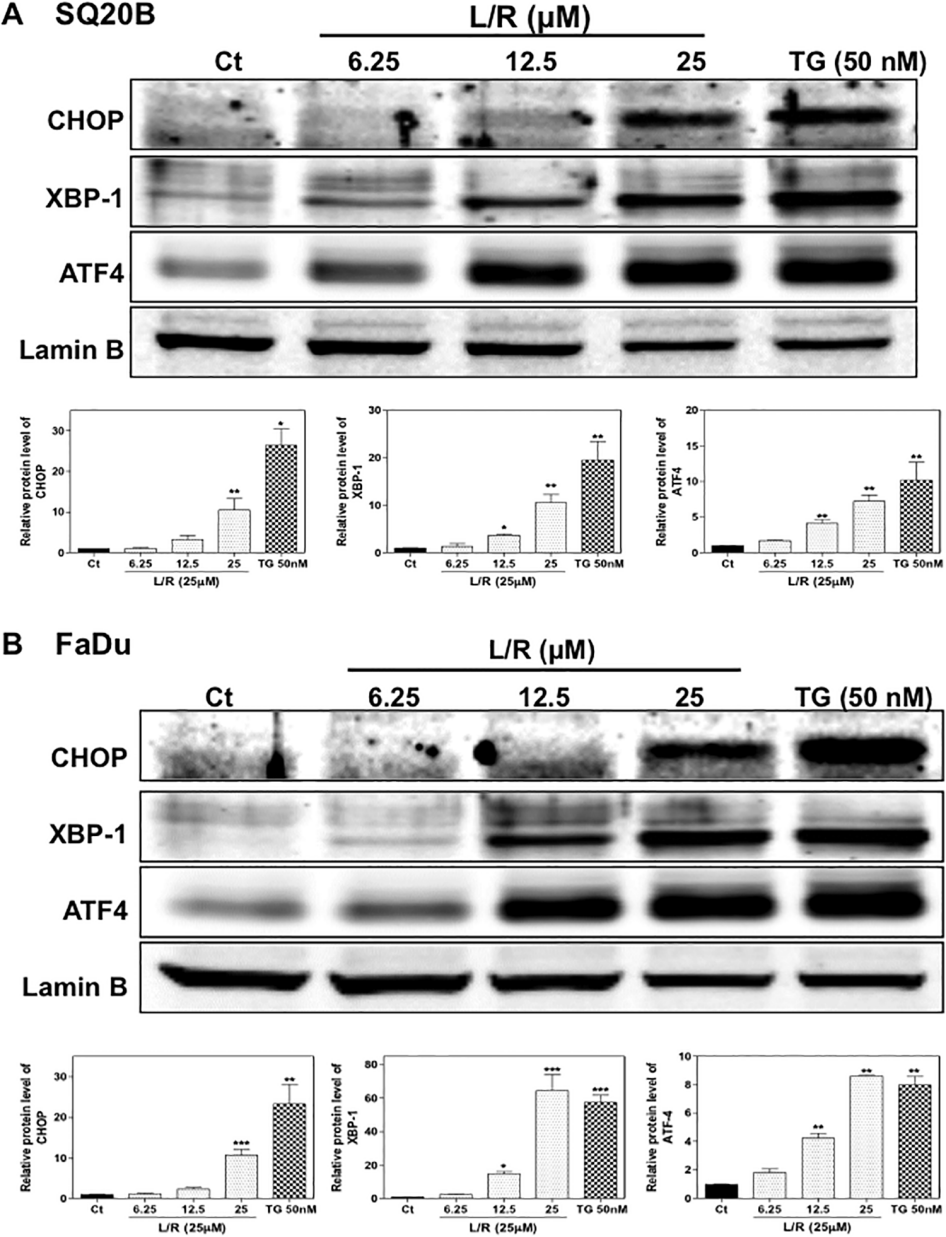
Effect of HIV PIs on protein levels of the UPR genes in HNSCC. HNSCC cells, SQ20B and FaDu, were cultured in complete medium and treated with different concentrations of L/R (0, 12.5, 25, and 50 μM) or TG (50 nM) for 24h. At the end of treatment, nuclear proteins were isolated. The protein levels of CHOP, ATF-4, XBP-1 and lamin B were determined by Western blotting analysis. Lamin B was used as a loading control. Representative images of immunoblots are shown. Values are mean ± S.E. of three independent experiments. Statistical significance relative to vehicle control, *P<0.*05*; **P<0.*01*. (A) SQ20B cells; (B) FaDu cells.

**Fig 5:**
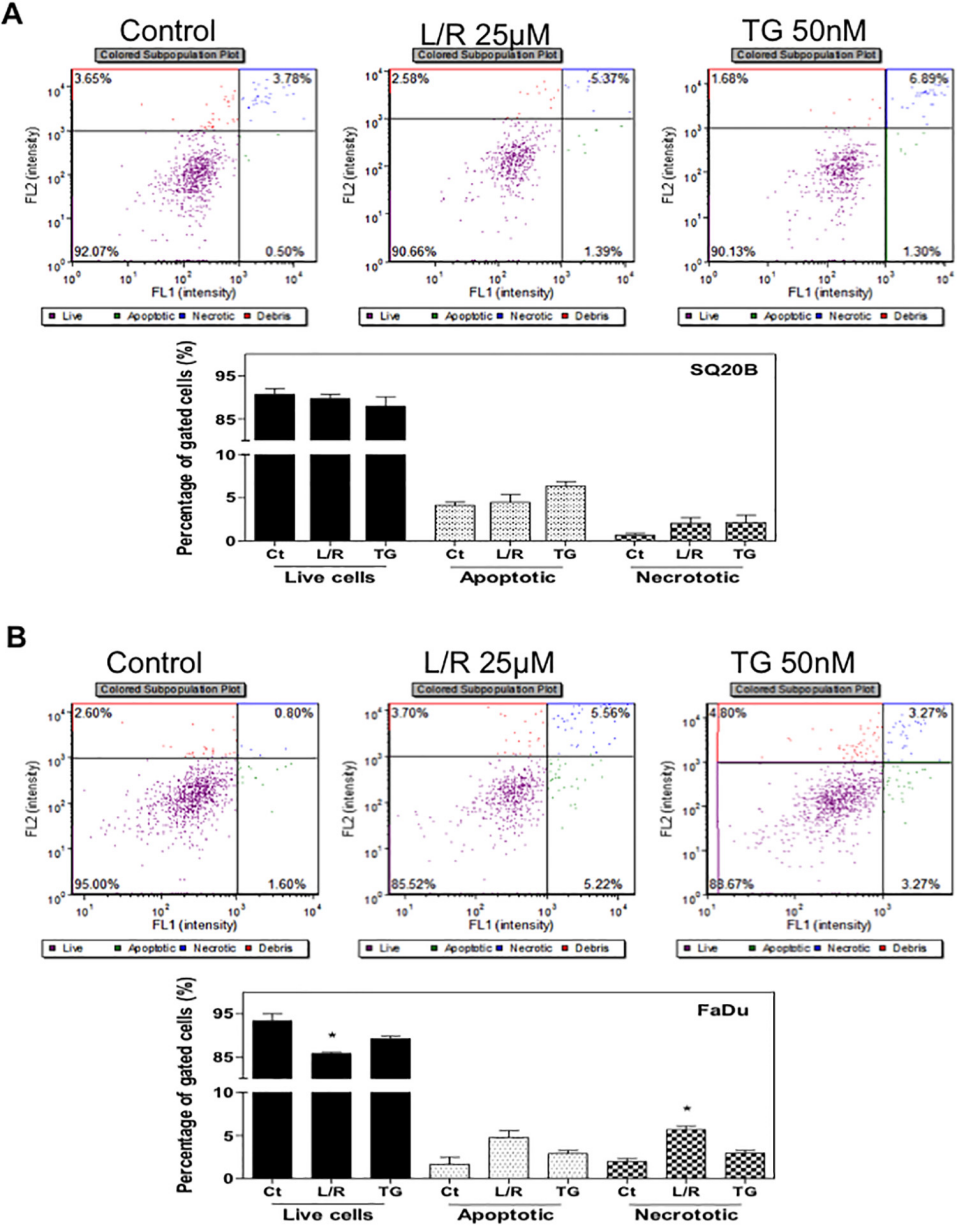
Effect of HIV PIs on apoptosis in SQ20B and FaDu cells. Human HNSCC cells SQ20B and FaDu were cultured in complete medium and treated with HIV PIs L/R (25μM) or TG (50 nM) for 24h. At the end of treatment, cells were stained with Annexin V- PI and analyzed using the Cellometer Vision CBA system. FL1 represents Annexin V staining for apoptotic cells and FL2 represents PI staining for necrotic cells. Representative images of flow cytometry analysis and percentages of apoptotic and necrotic cells from three independent experiments are shown: (A) SQ20B; (B) FaDu. Statistical significance relative to vehicle control, *P<0.*05*.

Our previous studies have shown that HIV PIs induce ER stress and cell apoptosis in macrophages, hepatocytes, intestinal epithelial cells, and adipocytes [16, 18, 19, 34]. To examine whether L/R also activates ER stress in HNSCC cells, we first examined the effect of L/R on mRNA expression of the key genes involved in the UPR. As shown in Fig 3, L/R dose-dependently increased CHOP, ATF-4, and XBP-1 mRNA levels in both SQ20B and FaDu cells. TG was the positive control for ER stress induction. As shown in Fig 4, L/R-induced mRNA expression of the UPR genes was correlated to up-regulation of the corresponding protein level. L/R had a more profound effect on ATF-4 expression. To further determine whether L/R-induced UPR activation was able to promote cell apoptosis, Annexin V-PI staining was used to detect apoptotic and necrotic cells after treatment with L/R (25 μM) or TG (50 nM) for 24 h. As shown in Fig 5, L/R alone did not induce significant apoptosis in SQ20B and FaDu cells. However, pre-treatment with L/R markedly increased radiosensitivity of both HNSCC cell lines. Compared to the control group, L/R reduced cell viability by 60% (data not shown).

### Effect of HIV PIs on HNSCC cell migration

**Fig 6:**
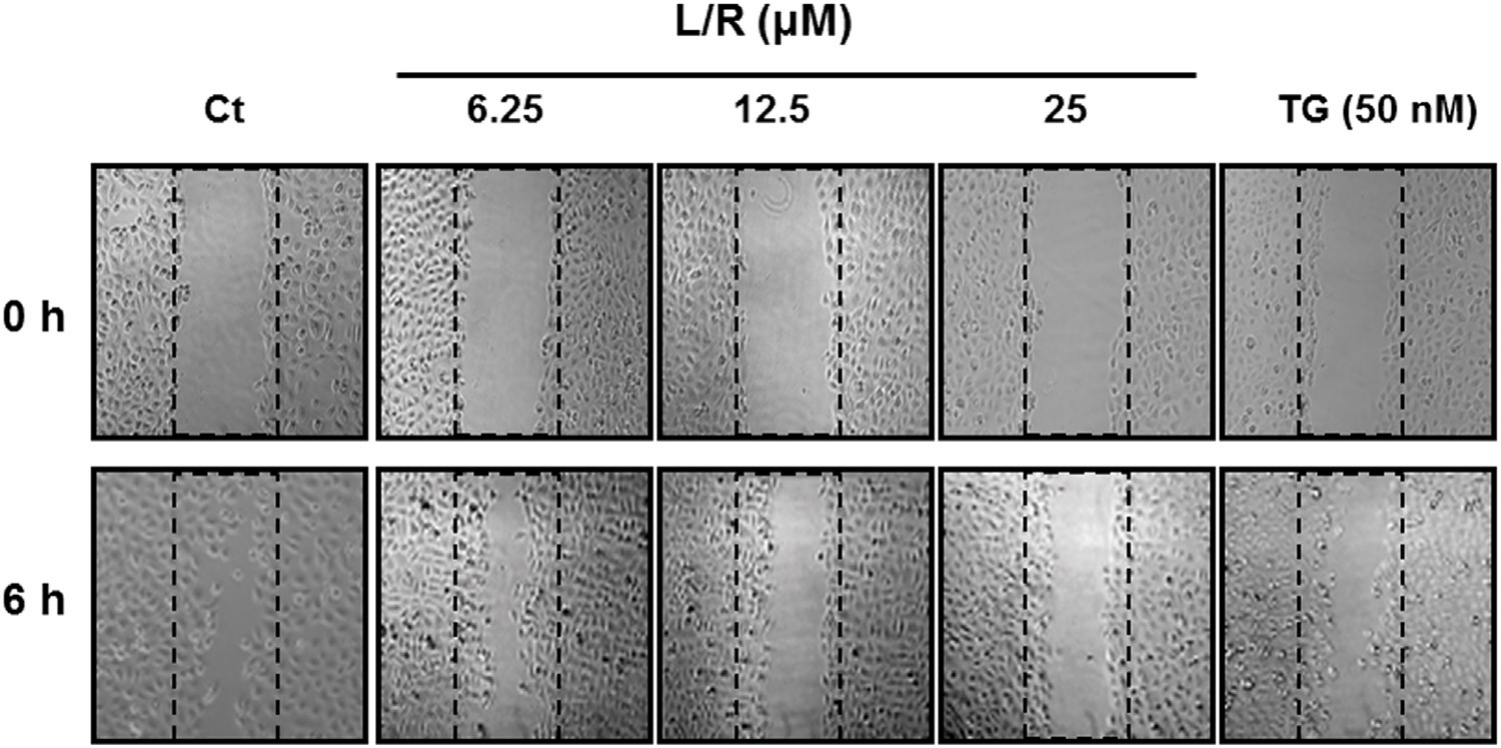
Effect of HIV PIs on cell migration. HNSCC SQ20B cells were plated on 12-well plates until confluent. Cells were scratched to simulate a wound and images were recorded as “0h”. Cells were treated with different amounts of HIV PIs (L/R, 0–25 μM) for 6 h. The images of wound areas were recoded as described in Methods. Representative images are shown.

Cancer cells spread from the initial site of tumor growth by first invading the surrounding tissue (migration). The ability of tumor cells to migrate is closely linked to tumor growth and invasiveness. To determine the effect of HIV PIs on HNSCC cell migration, we did a wound healing assay in SQ20B cells as described in Methods. As shown in Fig 6, L/R dose-dependently inhibited cell migration. Similar results were obtained with TG, the ER stress inducer.

### Effect of HIV PIs on cell cycle distribution

**Fig 7:**
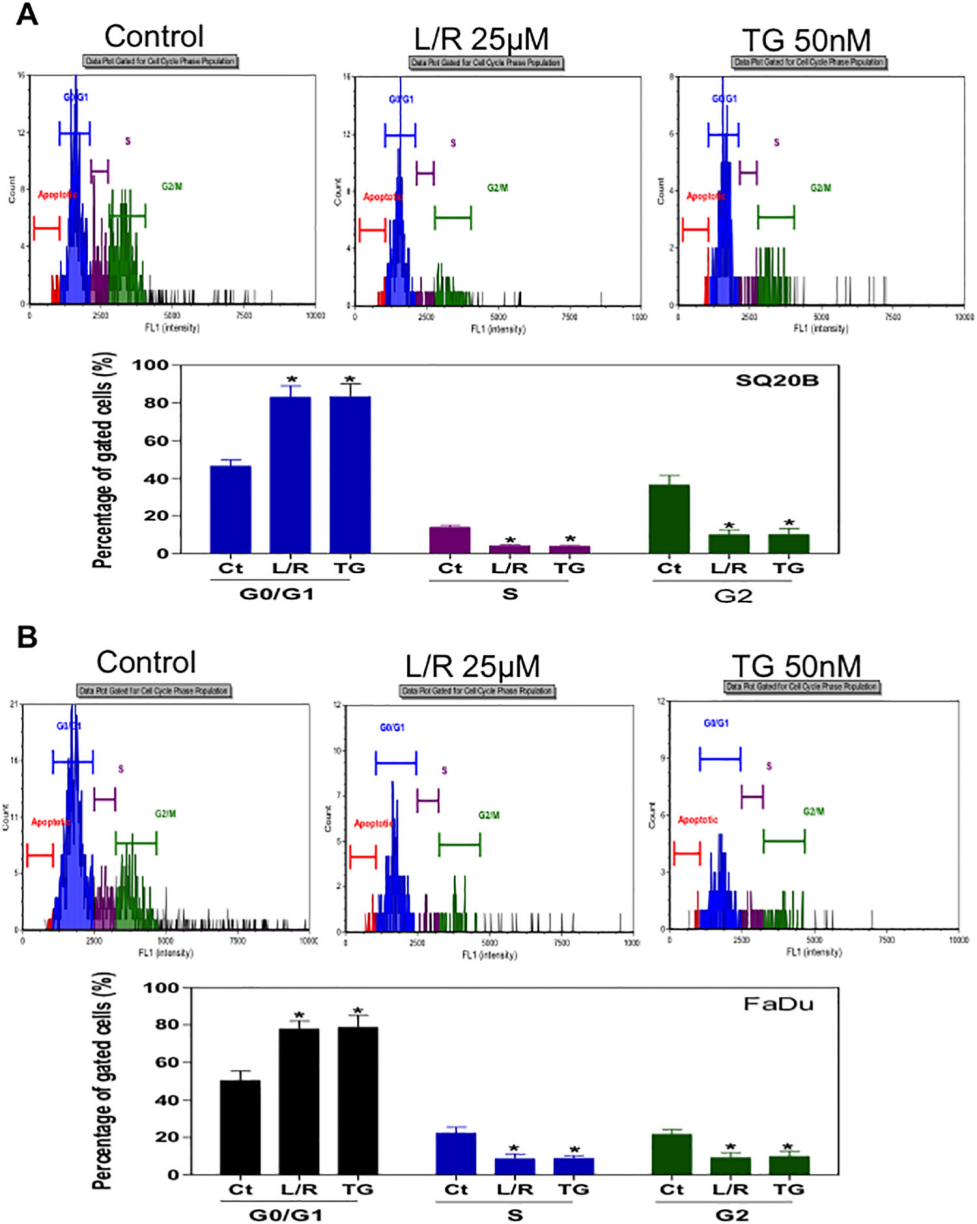
Effect of HIV PIs on cell cycle distribution of HNSCC cells. Human HNSCC cells (A) SQ20B and (B) FaDu were cultured in complete medium and treated with L/R (25μ M) or TG (50 nM) for 24h. At the end of treatment, cells were stained with the Cellometer PI Cell Cycle kit and analyzed by the Cellometer Vision CBA system. Cell population percentages of G_0_/G_1_, S and G_2_/M phases are shown. Values of each group are mean ± S.E. of three independent experiments. *P<0.*05*, Statistical significance relative to control group.

In order to identify the potential mechanisms underlying HIV PI-induced radiosensitivity of HNSCC, we examined the effect of L/R on cell cycle distribution. After treatment with L/R (25 μM) or TG (50 nM) for 24h, cells were analyzed using the Cellometer PI Cell Cycle Kit from Nexcelom. As shown in Fig 7, L/R significantly increased SQ20B and FaDu cells at G0/G1 phase and cells at S/G2 phases were markedly decreased. These results indicated that L/R increased radiosensitivity of SQ20B and FaDu cells through inducing G_0_/G_1_ phase arrest rather than promoting apoptosis.

Overexpression of EGFR is detected in 90% of HNSCC. EGFR-mediated activation of PI3K/Akt and mitogen-activated/extracellular signal-regulated kinase (MEK) contributes to activation of NF-ƙB and induction of inflammatory response. Activation of Akt also induces activation of the mammalian target of rapamycin (mTOR). Emerging evidence indicates that phosphorylated Akt plays an important role in radio-resistance in cancer cells [37–39]. It also has been reported that the HIV PI, nelfinavir, has an inhibitory effect on Akt activation and mTOR [26, 27]. To determine whether L/R have any effect on Akt activation in HNSCC, we measured the phosphorylation level of Akt in SQ20B and FaDu cells after treatment with L/R for 24 h by western blot analysis. The results indicated that L/R had no significant effect on Akt activation in HNSCC (Data not shown).

### Effect of HIV PIs on the expression of cyclin D1

**Fig 8:**
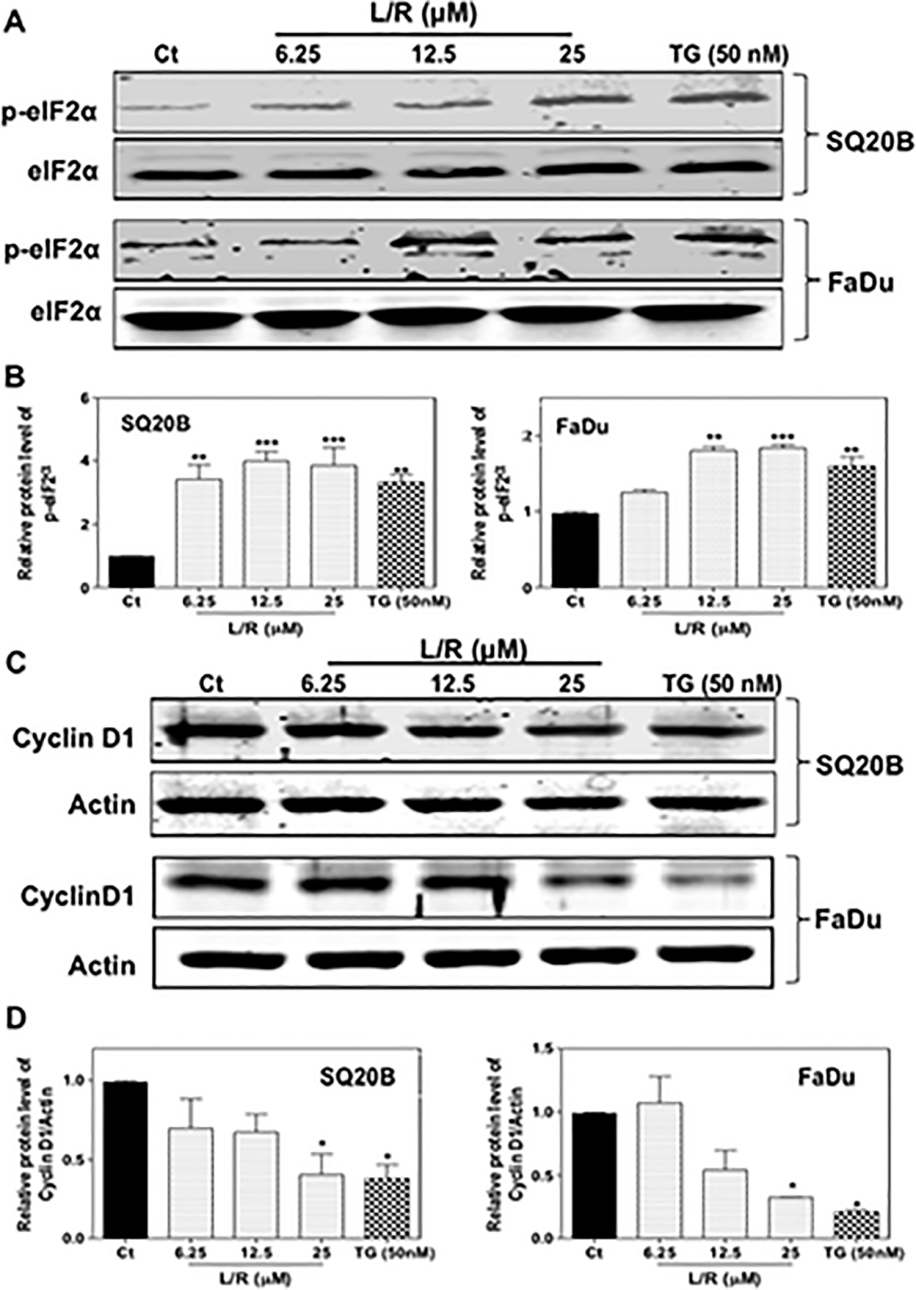
Effect of HIV PIs on PERK/eIF2α activation and cyclin D1 expression in HNSCC cells. Human HNSCC cells, SQ20B and FaDu, were cultured in complete medium and treated with different concentrations of L/R (0, 12.5, 25, and 50 μM) or TG (50 nM) for 24h. At the end of treatment, total cellular proteins were isolated. The protein levels of p-eIF2α, eIF2α, cyclin D1, and β-actin were determined by Western blotting analysis. β-actin was used as a loading control. Representative images of immunoblots are shown. Values are mean ± S.E. of three independent experiments. Statistical significance relative to vehicle control, *P<0.*05*; **P<0.*01*. (A) Protein levels of p-eIF2α and eIF2α; (B) Protein levels of cyclin D1.

HNSCC exhibits increased expression of cyclin D1, a key moderator in cell cycle progression from G_1_ phase to S phase. Overexpression of cyclin D1 correlates to poor prognosis of HNSCC [40]. It has been shown that ER stress-mediated activation of the PERK/eIF2α/ATF-4 signaling pathway down-regulated expression of cyclin D1 by suppressing global protein synthesis. To further identify the mechanism underlying HIV PI-induced cell cycle arrest in HNSCC, we examined the effect of HIV PIs on PERK/eIF2α activation and cyclin D1 expression. As shown in Fig 8A and 8B, L/R dose-dependently increased the protein levels of phosphorylated eIF2α (p-eIF2α) in both SQ20B and FaDu cells. Furthermore, L/R-induced increase of p-eIF2α was correlated to the decrease of cyclin D1 protein levels (Fig 8C), suggesting the contribution of activation of ER stress to HIV PI-enhanced radiosensitivity of SQ20B and FaDu cells.

## Discussion

HNSCC is still a devastating malignancy worldwide. The overall five-year survival rate is only about 50% despite recent advances in therapy. Radiotherapy is the cornerstone of current therapy for HNSCC. The major challenge to the management of HNSCC is the rapid development of resistance to radiotherapy [41]. HIV PIs have been successfully used to inhibit HIV replication and have significantly reduced the morbidity and mortality of HIV patients in the past two decades. Several studies have reported that HIV PIs, such as nelfinavir, can increase the radiosensitivity of certain cancer cells including HNSCC [20–23, 27, 42–44]. However, the toxicity of nelfinavir is relatively higher than other HIV PIs and the underlying mechanisms still remain unknown.

In the present study, we investigated the effects of the most commonly used HIV PIs, L/R, on cell proliferation, migration, and radiosensitivity of HNSCC and further identified the potential mechanisms. The results indicated that L/R significantly inhibited cell growth and migration, and markedly enhanced radiosensitivity. Previous studies from our groups and others’ suggested that HIV PIs induce ER stress and apoptosis in different types of cells [18–20, 45]. It has been reported that ER stress sensitizes cancer cells to radiation [46, 47]. Although L/R did not directly induce apoptosis in HNSCC cells, radiosensitivity was significantly enhanced after pretreatment with L/R. Several studies have reported that nelfinavir sensitized HNSCC cells to radiation mainly by inhibiting the PI3/Akt signaling pathway [27]. The Ras/PI3K/Akt signaling pathway is considered to be an important radio-resistance pathway in cancer cells because of modulation of cell cycle progression, especially at the G_1_/S transition, and DNA damage repair after radiation exposure [24, 37–39, 48]. Cells under G_1_ phase prepare proteins and enzymes for the following DNA synthesis in S phase. The formation of two critical complexes, Cyclin D1/CDK4 and Cyclin E/CDK2, is the core mechanism of cell cycle progression from G_1_ to S phase. Akt is an important regulator of the activation of cyclin D1 and inhibition of P27kip1, a suppresser of the cyclin D1/CDK4 complex, and induces cell proliferation by promoting cells to pass through the G_1_ checkpoint [49, 50]. In addition, Akt also increases cancer cell survival after radiation by helping to repair radiation-induced DNA damage [51]. These findings suggested that targeting Akt represents a promising new anti-cancer therapy. However, inhibition of the Akt-signaling pathway alone is not enough to enhance radiosensitivity of HNSCC [27]. In contrast to nelfinavir, which required longer (3-day) treatment, L/R only required a shorter (1-day) treatment to enhance radiosensitivity of HNSCC cells. Also, L/R had no effect on Akt activation, suggesting that another Akt-independent pathway is involved in L/R-mediated radio-sensitization.

ER stress-mediated activation of the UPR has been implicated in cell growth arrest and inhibition of cell migration. In the current studies, we identified that L/R significantly activated the IRE1α/XBP1 and PERK/eIF2α/ATF4 in SQ20B and FaDu cells. Although L/R significantly up-regulated CHOP, a proapoptotic factor, there was no significant change in terms of cell apoptosis. These results suggest that an L/R-mediated increase of radiosensitivity of HNSCC cells is independent of suppression of Akt activation and induction of apoptosis.

Regulation of cell cycle checkpoints is important for cell growth. Our results indicated that L/R significantly arrested SQ20B and Fadu cells at the G_0_/G_1_ phase. Previous studies have shown that ER stress-mediated activation of PERK/eIF2α was correlated to the down-regulation of cyclin D1 by global translation suppression [52, 53]. Overexpression of PERK also induced significant reduction of CDK2 activity [53]. In addition, crosstalk between PERK-induced eIF2α phosphorylation and cyclin D1 protein degradation was reported recently [54]. Our studies also showed that L/R-mediated activation of the PERK/eIF2α pathway was linked to G_0_/G_1_ cell cycle arrest and down-regulation of cyclin D1 protein levels in SQ20B and FaDu cells. During the last decade, several chemotherapeutic agents, which induce cell cycle arrest, such as flavopiridol and paclitaxel, have been used to enhance radiosensitivity of cancer cells [55]. Our studies suggest that the HIV PIs, L/R, can be used to treat HNSCC in combination with other chemotherapies and radiotherapy.

## Conclusions

**Fig 9:**
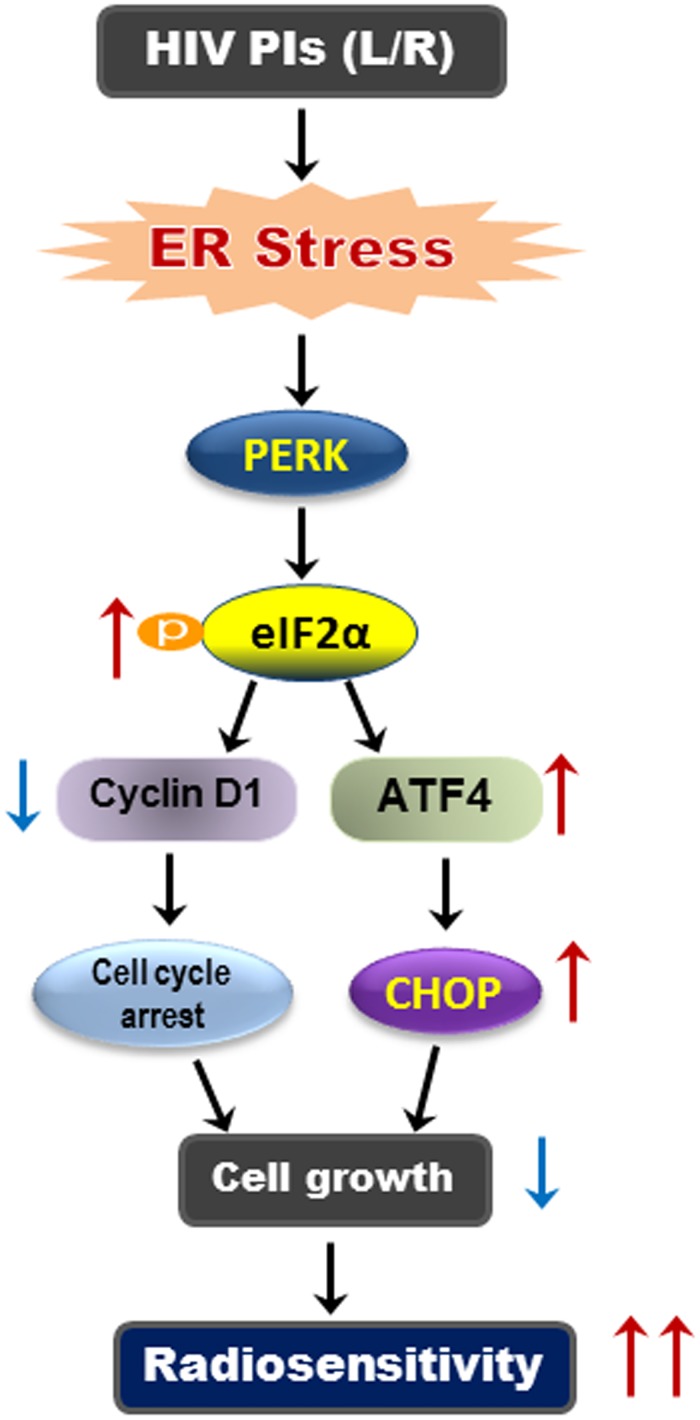
Proposed mechanism of HIV PI-induced radiosensitivity in HNSCC cells. HIV PIs induce ER stress and activate the UPR in HNSCC cell. Activation of PERK/eIF2α/ATF-4 will repress global protein translation, reduce cyclin D1 protein level and induce cell cycle arrest. ATF-4 also induces the expression of CHOP, which will inhibit cell growth. PERK, double-stranded RNA-activated protein kinase-like ER kinase; CHOP is also called GADD153 (growth arrest and DNA damage-inducing gene).

In summary, our study demonstrated that administration of HIV PIs (L/R) significantly enhanced radiosensitivity of SQ20B and FaDu cells. As illustrated in Fig 9, L/R induced activation of the ER stress response and subsequently activated PERK/eIF2α/ATF-4 and reduced cyclin D1 protein levels. L/R induced cell cycle arrest mainly through the inhibition of global protein translation and cell growth. Our studies suggest that activation of ER stress response represents one of the key mechanisms underlying HIV PI-induced radiosensitivity. This study provides a rationale for clinical investigation of the combination therapy using the FDA-approved HIV PIs (L/R) with radiotherapy in patients with HNSCC.
